# Disclosure of research results: a randomized study on GENEPSO‐PS cohort participants

**DOI:** 10.1111/hex.12390

**Published:** 2015-07-23

**Authors:** Julien Mancini, Elodie Le Cozannet, Anne‐Déborah Bouhnik, Noémie Resseguier, Christine Lasset, Emmanuelle Mouret‐Fourme, Catherine Noguès, Claire Julian‐Reynier

**Affiliations:** ^1^ Aix Marseille UniversitéUMR_S912IRDSESSTIMMarseilleFrance; ^2^ INSERMUMR912 (SESSTIM)MarseilleFrance; ^3^ APHMHôpital de la TimoneBiosTICMarseilleFrance; ^4^ Centre Léon BérardLyonFrance; ^5^ Institut CurieHôpital René HugueninSaint‐CloudFrance; ^6^ Institut Paoli‐CalmettesMarseilleFrance

**Keywords:** *BRCA1/2*, cohort, disclosure, research results, randomized controlled trials, satisfaction

## Abstract

**Background:**

There exist no recommendations as to how aggregate research results should best be disclosed to long‐term cohort participants.

**Objective:**

To study the impact of cohort results disclosure documents of various kinds on participants’ satisfaction.

**Design:**

Randomized study with a 2x2 factorial design.

**Setting and participants:**

The GENEPSO‐PS cohort is used to study the psychosocial characteristics and preventive behaviour of both *BRCA1/2* carriers and non‐carriers; 235 participants wishing to receive ‘information about the survey results’ answered a self‐administered questionnaire.

**Interventions:**

The impact of providing the following items in addition to a leaflet about aggregate psychosocial research results was investigated (i) an up‐to‐date medical information sheet about *BRCA1/2* genetic topics, (ii) a photograph with the names of the researchers.

**Main outcome measures:**

Satisfaction profiles drawn up using cluster analysis methods.

**Results:**

Providing additional medical and/or research team information had no significant effect on satisfaction. The patients attributed to the ‘poorly satisfied’ group (*n* = 60, 25.5%) differed significantly from those in the ‘highly satisfied’ group (*n* = 51, 21.7%): they were younger [odds ratio (OR) = 0.96, 95% confidence interval (0.92–0.99), *P* = 0.028], less often had a daughter [OR = 4.87 (1.80–13.20), *P* = 0.002], had reached a higher educational level [OR = 2.94 (1.24–6.95), *P* = 0.014] and more frequently carried a *BRCA1/2* mutation [OR = 2.73 (1.20–6.23), *P* = 0.017].

**Conclusions:**

This original approach to disclosing research results to cohort participants was welcomed by most of the participants, but less by the more educated and by *BRCA1/2* carriers. Although an easily understandable document is necessary, it might also be worth providing some participants with more in‐depth information.

## Introduction

Most previous research on the information conveyed to cohort participants has focused on individual results and incidental findings.^1^, ^2^, ^3^ Although the disclosure of aggregate research results has been investigated in depth on randomized controlled trials participants for the last 15 years,^4^ less information is available on cohort participants, where returning aggregate research results can also maintain trust in researchers and the scientific endeavour and be said to be an ethical duty.^5^, ^6^ Although ‘between‐wave’ messages including feedback leaflets are sometimes mailed to participants because they are expected to prevent attrition,^7^ the final aggregate results obtained are not systematically disclosed, and no recommendations are available as to how this ethical duty should best be performed. The aim of this study was therefore to investigate the topic of disclosure of aggregate research results more closely.

For more than 5 years, we have been following an on‐going French national cohort of *BRCA1/2* carriers/non‐carriers from families where a mutation has been identified, with a view to determining their psychosocial characteristics and their preventive behaviour (GENEPSO‐PS).^8^, ^9^, ^10^, ^11^, ^12^, ^13^ In the 5‐ and 10‐year follow‐up questionnaires, we asked the participants whether they would be interested in obtaining the research results, and the overall majority responded in the affirmative. In the framework of a mixed‐methods research design, in‐depth interviews were first conducted to determine whether participants wished to be given more information.^14^ These interviews showed that most of the GENEPSO‐PS cohort participants were interested in receiving the overall cohort results, but that they had no particular expectations *a priori*. At the time of the interview, they said they were simply glad to be in contact with the research team and to know who was ‘behind the questionnaires’.^14^ Carriers and non‐carriers of *BRCA1/2* mutations both said that they would like to be given medical findings about *BRCA1/2,* assuming the researchers to be a good source of the latest medical findings about their own or their familial condition. Despite their long‐term participation in the cohort, they were not sure what kind of results had been generated by their responses to the multiple follow‐up questionnaires. They also mentioned their wish to obtain information via regular postal mail because the cohort questionnaires had been delivered in this way.

The aim of the subsequent quantitative phase of the study, which corresponds to the results presented here, was to investigate the impact of including an up‐to‐date medical information sheet with the document presenting the aggregate psychosocial research results. The effects of a more personalized leaflet, including a photograph of the research team involved in GENEPSO‐PS and their names, were also tested using a 2x2 factorial design. The secondary objective was to determine the respondents’ satisfaction profiles and the corresponding individual characteristics.

## Materials and methods

### Participants

The GENEPSO‐PS cohort is used to study the psychosocial characteristics and preventive behaviour of both *BRCA1/2* carriers and non‐carriers. All participants were aware of their mutational status. They had completed a baseline questionnaire before disclosure of the *BRCA1/2* genetic test results and received other questionnaires at disclosure and then 6 months, 1 year, 2 years, 5 years and 10 years later. At the time of the study, the median follow‐up of participants was 10 years (range 7–13 years). Among the 454 participants who answered the 5‐ or 10‐year questionnaire, 345 (76%) stated that they wished to receive ‘information about the survey results when available’. All the participants interested in receiving psychosocial research results were subsequently randomized using a 2x2 factorial design to study the impact of cohort results disclosure documents of various kinds. Participants belonging to the same family were all assigned to the same group in order to prevent the occurrence of a contamination bias. The size of the sample corresponded to the number of members of the cohort who expressed the wish to receive information about the results. However, we expected to be able to compare subgroups consisting of at least 130 patients, and therefore to detect medium to small effect sizes (0.35) with a power of 80%.^15^

### Randomized interventions

**Figure 1:**
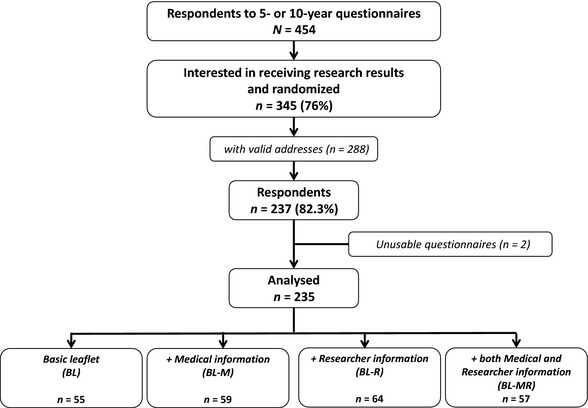
Participants’ flow diagram.

All the participants were randomly assigned to one of the four intervention arms (Fig. 1):

1. Basic leaflet (BL): participants assigned to this group received only the original leaflet presenting the main psychosocial research results obtained on the GENEPSO‐PS cohort. This was a simply written coloured double‐sided A3 format document issued in January 2013. It included some general information about the recruitment of the cohort and the funders of the project, as well as the main findings published in the literature, and were illustrated by some anonymous statements made by cohort participants. The main results obtained focused on the following points: respondents’ perceptions of health research questionnaires,^9^ the cancer risk management strategies adopted by *BRCA1/2* carriers and non‐carriers,^8^, ^13^ their theoretical intentions about having preimplantation genetic diagnosis and prenatal diagnosis,^10^ the patterns of disclosure of genetic test results to employers by unaffected carriers^11^ and the psychological impact of genetic testing.^12^
2. Medical information (BL‐M): participants in this group received the basic leaflet as well as an extra biological and medical information sheet about *BRCA1/2* genetic topics. In short, it explained that more than 2000 *BRCA1/2* gene mutations have been discovered, that annual MRI screening is now recommended for *BRCA1/2* carriers over 30 years of age and that risk‐reducing mastectomy and oophorectomy can prevent more than 90% of the cases of breast and ovarian cancer which occur, respectively. Information about the genetic counselling activities in France was also provided.
3. Information about the research team (BL‐R): participants in this group were given a more personalized version of the basic leaflet, with a photograph of the research group giving the researchers’ names. The aim here was to make the survey based on anonymous mailed questionnaires less impersonal.
4. Medical information plus Researchers’ identity (BL‐MR): participants assigned to this group received both the personalized version of the leaflet and the extra sheet giving medical information about *BRCA1/2*.

These documents were written by two of the authors (CJR and NR) and improved at a workshop involving all the co‐authors. A second version was issued and discussed by phone with three of the cohort participants, who had agreed at the qualitative interviews described elsewhere^14^ to assess the content of this document. The third and final version was subsequently submitted to the working group consisting of the co‐authors, and the researchers involved in the preliminary qualitative stage of the project. During the last stage, the final draft (including photographs and icons) was submitted to a communication agency (septLieux.com), which edited the final version of the leaflet. The whole document production process took 7 months. The final, most complete version of the document (BL‐MR arm) is provided under the heading Appendix S1.

### Measures

Participants’ assessment of the disclosure document was measured using a special self‐administered questionnaire accompanying the document. This questionnaire was sent in April 2013 to all the randomized participants. Two reminders were sent later on, in May (a letter alone) and June 2013 (a letter and the questionnaire).

#### Assessment of the document

After checking that the document provided has been read (totally, partly or not yet), respondents’ assessment of the documents was determined using nine questions. First, four numerical scales (ranging from 0: ‘not at all’ to 10: ‘completely’) were used to ascertain how useful, interesting and understandable the document was and whether it met participants’ expectations. Secondly, all the participants were asked three questions about whether the amount of information (general information, medical information and information about the research team) provided in the disclosure document was sufficient. The last two questions were intended to determine whether the patients found the document reassuring and whether it had changed (increased/decreased) their trust in research or not. Except for the four numerical scales, the answers were all given on 5‐point Likert scales, which were dichotomized secondarily to describe the number of participants who gave the answers ‘(very) insufficient’ information or ‘reassuring’ or ‘increasing’ trust in research.

#### Trust in medical researchers

A specific standardized four‐item questionnaire was used to assess the participants’ trust in medical researchers (TMR).^16^ The standardized score ranged from 0 to 10: the higher the score, the greater the trust.

#### Sociodemographics

These data included age, gender, living with a partner, offspring, educational level and occupation.

#### Medical characteristics

Only *BRCA1/2* mutation status and incident cancer diagnosis were used in this study.

#### Psychological characteristics

Physical and mental health‐related quality of life was evaluated using the Social Functioning (SF)‐12 Health Survey, a short version of the SF‐36 Health Survey.^17^

Participants’ depressive symptomatology was measured using the Center for Epidemiologic Studies – Depression (CES‐D) scale.^18^ Participants were subsequently classified using a binary variable: depressive symptoms/states were defined as CES‐D scores of ≥16 in the case of men and ≥23 in that of women.^19^

Information‐seeking behaviour, that is the general wish for health‐related information, was assessed using the six‐item Extent of Information Desired (EID) scale.^20^

Breast cancer risk perception was self‐reported using a 5‐point Likert scale ranging from ‘null’ to ‘very high’.

### Statistical analysis

Univariate analyses were first performed to compare respondents and non‐respondents and to compare the randomized groups. Anovas and Student *t*‐tests were used to compare continuous data, and chi‐square tests were used on categorical data. Mann–Whitney tests (on continuous data) and Fisher's exact tests (on categorical data) were performed when appropriate.

Secondly, a binary logistic regression model was used to identify the factors independently associated with participants’ interest in disclosure of the results obtained on the cohort as a whole.

Thirdly, to define participants’ satisfaction patterns, the hierarchical cluster analysis method was used. With this exploratory method, participants can be classified in terms of their profiles; those falling in the same group are more similar to each other than to those in other groups. All the questions used to determine participants’ satisfaction with the document described above were used to define clusters. In addition to the broadly defined notion of ‘satisfaction’, TMR was also included because it was expected to reflect general satisfaction with the disclosure process, as opposed to specific satisfaction with the disclosure document. The clusters were defined using a Euclidian distance measure and Ward's dissimilarity methods.

Lastly, to study the factors potentially associated with the satisfaction profiles, multinomial logistic regression was performed using a backward elimination procedure. All the variables found in the univariate analyses to be associated with a *P*‐value <0.25 were tested in this model. First order interactions were systematically tested in the final model.

All the statistical analyses were two‐tailed, and the results were taken to be statistically significant when *P*‐values <0.05 were obtained. These analyses were performed using IBM SPSS Statistics 18.0 (IBM Inc., New York, NY, USA), except for the cluster analysis, which was performed with SPAD 3.21 (Coheris, Suresnes, France).

## Results

### Participants

Among the 454 eligible participants, as mentioned above, 345 (76.0%) expressed the wish to receive information about the results of the psychosocial study. Participants carrying a *BRCA1/2* mutation expressed greater interest in the research results than non‐carriers [adjusted odds ratio = 1.96; 95% confidence interval (1.20 to 3.23)]. Interested participants also had a higher educational level [adjusted odds ratio = 1.64 (1.0. to 2.60)] than the others. No other differences were observed in terms of the participants’ sociodemographic or psychosocial characteristics.

Among the 345 interested participants who were randomized after secondary exclusion of 57 participants who were no longer living at the address they had given, 235 questionnaires were available for analysis (response rate = 82.3%; Fig. 1). The median time elapsing as participants expressed their interest in receiving research results was 4 years, and no differences were observed in this respect between respondents and non‐respondents. Nor were there any differences between respondents’ and non‐respondents’ main characteristics (age; gender; *BRCA1/2* mutation status; living with a partner; offspring; educational level; depressive symptoms; cancer risk perception; and the type of document received). Respondents’ sociodemographic, medical and psychological characteristics are presented in Table 1. About half of them (48.9%) were *BRCA1/2* carriers.

**Table 1:** Respondents’ characteristics (*N* = 235)

		*n* a	%
Sociodemographic characteristics			
Mean age in years (SD)		51.5	(11.3)
Gender	Men	12	5.1%
	Women	223	94.9%
Living with a partner	Yes	185	80.1%
Number of children	0	7	3.2%
	1	34	15.5%
	2	104	47.5%
	3 and more	74	33.8%
Number of girls	None	83	35.3%
	At least one	152	64.7%
Educational level	<High school	95	40.4%
	≥High school	140	59.6%
Health‐care profession	Yes	52	22.1%
Medical characteristics			
* BRCA1/2* mutation carrier	Yes	115	48.9%
* *Incident cancer diagnosis during follow‐up	Yes	24	10.2%
Psychological characteristics			
* *Depressive symptoms (CES‐D^b^)	Yes	41	17.9%
* *Breast cancer risk perception	High/Very high	110	46.8%
* *Extent of Information Desired (EID), mean (SD)		16.4	(3.4)
* *Health‐related quality of life (SF‐12), mean (SD)	Physical (PCS)	52.6	(7.6)
	Mental (MCS)	44.2	(10.5)

a Data are *n* (%) unless otherwise specified.

b CES‐D: Center for Epidemiologic Studies on Depression (Depressive symptoms: ≥16 for men; ≥23 for women).

### Participants’ assessment of the document

Most of the participants had read the whole document or part of it (96.2%). On average, on a scale ranging from 0 to 10 (the highest level of approval), the document met the participants’ expectations (6.8, SD:2.2) and was thought to be useful (7.0, SD:2.1), interesting (7.3, SD:2.0) and understandable (8.7, SD:1.5). About one of every three participants (35.3%) stated that the amount of information provided in the disclosure document was insufficient. Average TMR was 7.5 (SD:1.4); 28.1% of the participants stated that the document had increased their trust in research; and 25.1% said that it was reassuring. Details of these responses are given in Table 2.

**Table 2:** Satisfaction profiles depending on the type of disclosure document received

	Additional medical information				*P* value	Additional researcher information				*P* value
	Without (BL + BL‐R) *n* = 119		With (BL‐M + BL‐MR) *n* = 116			Without (BL + BL‐M) *n* = 114		With (BL‐R + BL‐MR) *n* = 121		
	Mean	SD	Mean	SD		Mean	SD	Mean	SD	
Participants’ assessment of the document^a^										
Useful	6.9	2.0	7.0	2.2	0.834	7.0	2.2	6.9	2.1	0.562
Interesting	7.3	2.0	7.3	2.1	0.843	7.5	2.1	7.1	2.0	0.238
Understandable	8.6	1.6	8.8	1.4	0.318	8.9	1.5	8.6	1.5	0.178
Met participants’ expectations	6.5	2.2	7.0	2.1	0.138	6.9	2.2	6.6	2.1	0.277
Trust in Medical Researchers^a^	7.6	1.5	7.4	1.3	0.446	7.6	1.5	7.4	1.3	0.375

	*n*	%	*n*	%		*n*	%	*n*	%	
Insufficient information	45	37.8	38	32.8	0.417	39	34.2	44	36.4	0.730
Insufficient research group information	17	14.3	17	14.7	0.936	22	19.3	12	9.9	**0.041**
Insufficient Medical information	44	37.0	38	32.8	0.498	42	36.8	40	33.1	0.543
Reassuring information	31	21.6	28	24.1	0.735	27	23.7	32	26.4	0.626
Document increased trust in research	31	26.1	35	30.2	0.482	32	28.1	34	28.1	0.996

Intervention arms: BL = Basic leaflet; BL‐M = Medical (BL with additional medical information); BL‐R = Researchers (BL with additional information about the research team); BL‐MR = Medical Researchers (BL with additional medical information and information about the research team). Bold characters denote statistical significance.

a Ranging from 0 to 10, where 10 reflects greater appreciation or trust.

### Impact of the various disclosure documents

The four randomized groups were similar in terms of participants’ response rates, sociodemographic characteristics, *BRCA1/2* mutation status and psychological characteristics. Only the amount of information desired differed (EID scale, *P*‐value = 0.043), but when systematic adjustments for EID scale were made to account for this potential confounder, it did not affect the results of the comparative analyses presented in the Table 2 (data not shown).

Lastly, 116 participants received the leaflet containing extra medical information (BL‐M and BLM‐MR arms) and 121 participants received additional information about the research team (BL‐R and BLM‐MR arms, Fig. 1). Being given additional medical information and/or information about the research team had no effect on the participants’ appreciation levels (Table 2). Only participants who had received the document containing a photograph of the researchers and their names less frequently expressed the opinion that the amount of information about the research team was insufficient (9.9 vs. 19.3%, *P*‐value = 0.041).

### Satisfaction profiles

A 3‐cluster pattern of distribution emerged spontaneously from the cluster analysis performed using an automatic procedure (Table 3). Statistically significant differences were observed in all the variables chosen *a priori* for use in the clustering procedure.

**Table 3:** Satisfaction profiles obtained through cluster analysis

	All *N* = 235		‘Poorly satisfied’ *n* = 60		‘Averagely satisfied’ *n* = 124		‘Highly satisfied’ *n* = 51		*P* value
	Mean	SD	Mean	SD	Mean	SD	Mean	SD	
Participants’ assessment of the document^a^									
Useful	7.0	2.1	4.4	1.5	7.4	1.3	8.9	1.2	<0.001
Interesting	7.3	2.0	4.9	1.8	7.6	1.2	9.2	1.0	<0.001
Understandable	8.7	1.5	8.3	1.9	8.7	1.3	9.4	1.0	<0.001
Met participants’ expectations	6.8	2.2	4.2	1.4	7.1	1.3	9.0	1.2	<0.001
Trust in medical researchers^a^	7.5	1.4	6.9	1.4	7.6	1.3	8.0	1.3	<0.001

	*n*	%	*n*	%	*n*	%	*n*	%	
Insufficient information	83	35.3	46	76.7	32	25.8	5	9.8	<0.001
Insufficient research group information	34	14.5	18	30.0	15	12.1	1	2.0	<0.001
Insufficient medical information	82	34.9	44	73.3	35	28.2	3	5.9	<0.001
Reassuring information	59	25.1	7	11.7	21	16.9	31	60.8	<0.001
Document increased trust in research	66	28.1	3	5.0	25	20.2	38	74.5	<0.001

a Ranging from 0 to 10, where 10 reflects the highest appreciation or trust.

The first profile, which was composed of 25.5% of the sample, consisted of ‘poorly satisfied’ patients. The opposite profile was found in the third profile, which included 21.7% of the sample and consisted of ‘highly satisfied’ respondents. The remaining half of the study population (52.8%) corresponded to the second, ‘averagely satisfied’ profile.

### Factors associated with satisfaction profiles

In the univariate comparisons, the ‘highly satisfied’ participants were the oldest participants (Table 4): more than two‐thirds of them were non‐carriers of a *BRCA1/2* mutation and 86.3% had at least one daughter. In addition, 58.8% of them had a lower educational level. The ‘poorly satisfied’ participants were the youngest, about two‐thirds of them were *BRCA1/2* mutation carriers and almost three quarters (71.7%) of them had a higher educational level. None of the other sociodemographic, medical or psychological data collected differed between the profiles. Nor did the type of document received as the result of the randomization process differ significantly between the three profiles.

**Table 4:** Respondents’ characteristics vs. their satisfaction profiles

Sociodemographic characteristics	‘Poorly satisfied’ *n *= 60		‘Averagely satisfied’ *n *= 124		‘Highly satisfied’ *n* = 51		P value
	*n* a	%	n	%	n	%	
Mean age, in years (SD)	49.2	(9.6)	50.4	(10.6)	56.8	(13.4)	**<0.001**
Female gender	55	91.7%	120	96.8%	48	94.1%	0.323
Living with a partner	48	80.0%	98	79.7%	39	81.3%	0.973
Educational status ≥ High school	43	71.7%	76	61.3%	21	41.2%	**0.004**
Health‐care profession	14	23.3%	23	18.5%	15	29.4%	0.531
Number of children							
0	4	7.3%	3	2.6%	0	0.0%	0.197
1	7	12.7%	16	14.0%	11	22.0%	
2	27	49.1%	51	44.7%	26	52.0%	
3 and more	17	30.9%	44	38.6%	13	26.0%	
At least one daughter	34	56.7%	74	59.7%	44	86.3%	**0.001**
Medical characteristics							
* BRCA1/2* carrier	37	61.7%	60	48.4%	18	35.3%	**0.021**
Cancer comorbidity	4	6.7%	12	9.7%	8	15.7%	0.283
Psychological characteristics							
Depressive symptoms (CES‐D)	14	24.1%	20	16.4%	7	14.3%	0.340
High/very high breast cancer risk perception	30	50.0%	62	50.0%	18	35.3%	0.177
Extent of Information Desired (EID), mean (SD)	16.5	(3.3)	16.3	(3.5)	16.7	(3.5)	0.709
Health‐related quality of life (SF‐12), mean (SD)							
Physical (PCS)	53.1	(6.7)	52.4	(7.5)	52.3	(9.0)	0.836
Mental (MCS)	43.0	(10.9)	44.3	(10.1)	45.6	(10.9)	0.445
Content of document (randomized)							
Additional Medical information	30	50.0%	56	45.2%	30	58.8%	0.258
Additional Research team information	27	45.0%	72	58.1%	22	43.1%	0.101

Bold characters denote statistical significance.

a Data are *n* (%) unless otherwise specified.

The associated factors tested in the univariate comparisons were again found to be significant in the multinomial logistic regression model (Table 5). The members of the ‘poorly satisfied’ group differed significantly from the members of the ‘highly satisfied’ (reference) group in that they were younger, less often had at least one daughter, had reached a higher educational level and more often carried a *BRCA1/2* mutation. The ‘averagely satisfied’ participants differed significantly from the ‘highly satisfied’ ones in that they were younger and less often had at least one daughter. None of the first order interactions tested were significant.

**Table 5:** Factors independently associated with satisfaction profiles (multinomial logistic regression; reference group = ‘Highly satisfied’ respondents)

	Poorly (vs. Highly) satisfied			Averagely (vs. Highly) satisfied		
	OR	95% CI	*P* value	OR	95% CI	*P* value
Age, per 1‐year increase	0.96	0.92–0.99	**0.028**	0.96	0.93–0.99	**0.021**
Without any daughters	4.87	1.80–13.20	**0.002**	4.28	1.73–10.58	**0.002**
Educational status ≥ High school	2.94	1.24–6.95	**0.014**	1.93	0.94–3.97	0.075
*BRCA1/2* mutation carrier	2.73	1.20–6.23	**0.017**	1.64	0.80–3.36	0.174

OR, adjusted odds ratio; CI, confidence interval

Bold characters denote statistical significance.

## Discussion

This is the first experimental study on the impact of documents of various kinds providing long‐term participants of a cohort survey with psychosocial epidemiological results. The results of this study show that the great majority of these participants wished to be informed about the aggregate research results obtained thanks to their participation and that they were satisfied on the whole with the document they were given. Providing a leaflet giving medical information and/or a more detailed description of the research group in addition to the basic document had no effect on the satisfaction scores recorded. However, the satisfaction profiles differed significantly depending on the participants’ characteristics: in particular, those more highly educated and those with a *BRCA1/2* mutation (GENEPSO‐PS cohort's exposed group) were the most interested *a priori* in obtaining the results, but they were also the least satisfied with the information provided. These results are worth discussing in detail.

The difference between the management of clinical trials and observational epidemiological studies is decreasing. It has been suggested, for example, that observational study protocols should be registered in the same way as interventional trials.^21^ Likewise, it is arguable on ethical grounds that research results should be shared with participants whatever the study design adopted. An increasingly large body of the literature has been devoted to the disclosure of aggregate and individual genetic results to biobank participants,^22^ but to our knowledge, few studies have focused so far on returning the aggregate cohort results obtained in epidemiologic studies to participants. Bunin *et al*.^23^ suggested back in 1996 that feedback letters should be a standard part of research protocols. Likewise, some professional guidelines have suggested that the results should be shared with participants. For example, the International Epidemiological Association guidelines for proper conduct in epidemiologic research recommend publishing ‘the main results in a form that reaches the participants of the study and other interested members of the community where the study took place – for example, in a newsletter, local newspapers’, etc. (cf. http://ieaweb.org/good-epidemiological-practice-gep/). Accordingly, most of the participants in the GENEPSO‐PS cohort surveyed here (76.0%) expressed the wish to be informed about the research results obtained.

As long‐term cohort participants are rarely informed about the aggregate research results obtained, no recommendations have been published so far about how this ethical obligation should be carried out. It was reported in a previous survey that the Internet was not the participants’ preferred mode of receiving the results of clinical trials^24^ because the mode of dissemination used was expected to be consistent with the mode of recruitment and/or that of the survey.^25^ As GENEPSO‐PS cohort participants’ consent and data are collected by postal mail, the same channel was used here to communicate the research results. A paper document was therefore prepared without any hyperlinks to sites providing further information. In order to tailor this document to participants’ wishes, a preliminary qualitative study was performed, in which participants’ expectations were investigated.^14^ Participants had no idea about what kind of results psychosocial questionnaires are liable to yield, and had made no attempt to find out, apart from stating at the interviews that they would like to be given medical findings about *BRCA1/2,* assuming the research team to be a good source of up‐to‐date medical information about their own or their familial condition.

Although participants expressed this wish at the interviews conducted in the qualitative study, the information provided in the extra biological and medical information sheet did not significantly increase their satisfaction or trust. They may have been disappointed by the fact that no major advances have been made in the field of prevention among *BRCA1/2* carriers. The information provided was expected to be fairly similar to that provided by the cancer geneticist at the time of *BRCA*1/2 testing, and the up‐to‐date information about the genetic counselling services available in France was not thought to be actionable. If we consider that participants might already knew there were no major medical advances, other explanations might be the wish for more in‐depth information, or the already high levels of satisfaction with the document provided (ceiling effect). Generally speaking, however, providing up‐to‐date medical information is not part of psychosocial researchers’ work, and it is not to be recommended as considerable resources can be required to review all the latest findings on a medical condition.

Adding a photograph of the research group and its members’ names increased the participants’ satisfaction with the information provided about the team, but it had no effect on the less specific indicators of satisfaction and did not increase participants’ trust in the researchers. However, a disclosure document also provides an opportunity of showing that a whole group of researchers is interested in analysing the responses collected. In a previous study performed in a different context, it was reported that an information sheet containing the photographs of the health‐care providers involved improved patients’ satisfaction with care.^26^ Those results are not directly comparable as patients might it find more useful to know more about their health‐care providers rather than about the researchers analysing their questionnaires. However, it highlights that the provision of photographs and explanations on the roles of persons involved can sometimes increase satisfaction towards communication skills and humanistic qualities. One of our future projects will consist in studying whether the GENEPSO‐PS participants’ overall satisfaction with the research group may predict the cohort's response rate to the next questionnaire.

Approximately, one‐quarter of the participants had a ‘poorly satisfied’ profile because they stated that they would have liked to be given more information in general and more medical information in particular (73%, Table 3). *BRCA1/2* carriers with a high educational level were the most interested in being given the results, but they were also the most disappointed with the document provided. Perhaps they were more informed and expected to receive more detailed information, in line with surveys showing that highly educated mothers participating in a longitudinal cohort study preferred to be given more detailed information about the biomarker results at disclosure,^27^ and that in comparison with the control subjects, the patients more frequently felt it was important to receive the results but were less satisfied and would have liked to be given more details.^23^ It is recommended and necessary to use lay language for this purpose and to check the readability of health information documents,^28^ but producing a single document which can be easily understood by all readers is probably not the right answer. Bunin *et al*.^23^ have suggested that several versions of a document could be tailored to meet the needs of readers with different educational levels. In addition, participants’ ‘exposed/unexposed’ status might have to be taken into account in longitudinal surveys. In the present case, the ‘unexposed’ participants, who were the *BRCA1/2* non‐carriers, were more often satisfied, possibly because the leaflet gave them some information about the potential psychosocial consequences of genetic testing in their family at a point when they had less opportunity of obtaining information about their familial mutation via the medical follow‐up procedures. Previous studies have also shown that patients tend to prefer being given individual results rather than aggregate study results.^22^ Participants who declared that they were interested in receiving the cohort study results may not have all taken the term ‘results’ to mean the same thing.^25^

Providing tailored information raises the issue of the time and resources which should be allocated by researchers to communicating results instead of working on new projects or writing scientific papers.^5^ People who would like to be given more detailed information could be given access to the relevant scientific publications. This would be easier than writing several disclosure documents and would also serve to acknowledge the fact that participants’ responses help to generate new scientific knowledge and teach people what the research process involves.^29^

The other factors independently associated with the satisfaction profiles were age and having at least one daughter. Except for the oldest patients (>80), age is known to be usually positively associated with participants’ satisfaction and should be taken into account when interpreting satisfaction data.^30^ However, this link may also reflect the reluctance of younger, more ‘connected’ people to read paper documents. The disclosure document was particularly highly appreciated by participants with daughters. Learning more about the average psychosocial impact of the decision to undergo genetic testing was probably reassuring to mothers, although we might have expected to find a positive interaction between having a daughter and *BRCA1/2* status. A written document can also fuel familial discussions,^31^ which might explain this increase in satisfaction.

This study has some limitations, however. A single disclosure document was tested on a selected population. The randomized patients did not all answer the questionnaire, but no significant differences were observed between respondents and non‐respondents. Satisfaction scales often show ceiling effects and a corresponding lack of discrimination. The approach used here yielded three distinct profiles, each corresponding to different participants’ characteristics.

This original attempt to disclose aggregate psychosocial research results to long‐term participants in an epidemiologic cohort met with the approval of most of the participants, especially older participants, those with a lower educational level, those with no *BRCA1/2* mutation and those with daughters liable to be concerned by genetic testing. This attempt to meet the wish for general medical information about the condition studied expressed by participants in the preliminary qualitative study did not significantly increase their satisfaction, and in view of this finding, this procedure should not be recommended for future use. The participants who were expected to be the most context‐specific health literates, because they were more educated and/or they were *BRCA1/2* carriers were less satisfied with the document provided. Although an easy‐to‐read document should be provided which can be understood by all the participants, the possibility of delivering more in‐depth information to the participants depending on their wish should be kept in mind. To achieve this objective and to promote active information‐seeking behaviour in some participants, it might also be worth giving them access to the relevant literature in the form of reprints of scientific papers, for example.

## Sources of funding

This study was funded by the French National Cancer Institute [INCa R08097AA/RPT08011AAA].

## Conflict of interest

None to declare.

## Supporting information


**Appendix S1.** GENEPSO‐PS cohort results disclosure document (BL‐MR arm version).
