# Supplementary material for: Disclosure of research results: a randomized study on GENEPSO‐PS cohort participants
Source: Health Expect. 2015 Jul 23;19(5):1023–35. doi: 10.1111/hex.12390 (PMC5054914; doi:10.1111/hex.12390)
Supplement: Supplementary file 1 — Appendix S1. GENEPSO‐PS cohort results disclosure document (BL‐MR arm version). [file HEX-19-1023-s001.pdf]

Résultats sur les aspects psychologiques

L'anxiété diminue progressivement après le résultat génétique

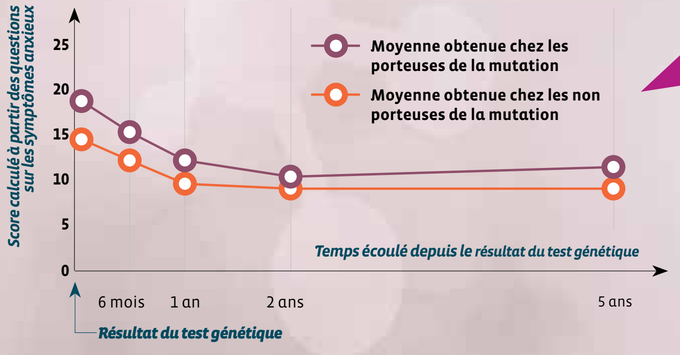

Le résultat génétique n'entraîne pas de dépression

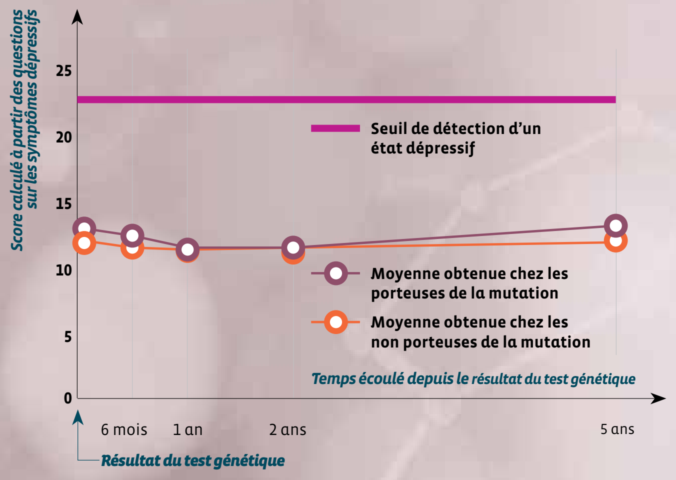

Marie (44 ans):
J'ai eu beaucoup de difficultés à accepter ce résultat dans les semaines qui ont suivi, mais grâce au soutien de ma famille et de mes meilleurs amis, j'ai pu progressivement prendre du recul et relativiser. Progressivement cet événement a cessé d'occuper la première place dans mes pensées et je retrouve foi dans l'avenir…

Dans les 6 mois suivant l'annonce du résultat génétique, vous en avez informé:

- pour plus de 9 personnes sur 10: votre conjoint, votre mère, votre(vos) sœur(s),
- pour plus de 8 personnes sur 10: votre père, votre(vos) frère(s), vos ami(e)s,
- pour plus de 6 personnes sur 10: votre(vos) enfant(s), la non-information étant due le plus souvent à un âge trop jeune.

Nathalie (44 ans):
J'en parle régulièrement avec mon mari et ma famille proche sans pour autant faire une fixation sur le cancer. Pour moi, savoir que j'ai plus de risque que les autres d'avoir le cancer du sein me permet de faire de la prévention et de me faire suivre régulièrement pour pouvoir réagir au moindre petit signe ou douleur…

Le programme GENEPSO:

Des participants, un réseau collaboratif de médecins (Groupe Génétique et Cancer), des chercheurs, associés pour un même objectif collectif de progression des connaissances.

Pour la recherche en Sciences Humaines et Sociales, des équipes de recherche coordonnées:

Par le Docteur Claire Julian-Reynier (Institut Paoli-Calmettes) & le Docteur Catherine Noguès (Institut Curie)

Des compétences de multiples disciplines de recherche:

Médecine, génétique, épidémiologie, biostatistiques, psychologie, anthropologie, économie, sociologie.

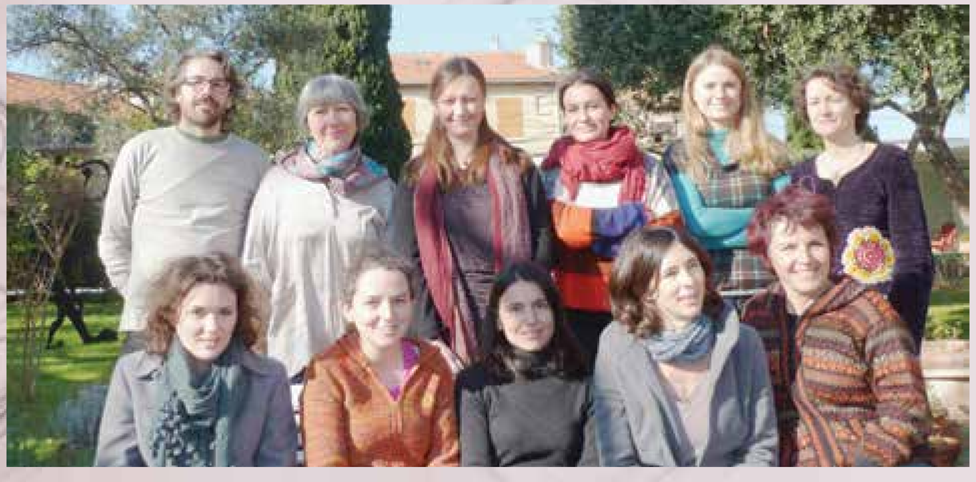

L'équipe de recherche au 25 janvier 2013

En haut, de gauche à droite: Julien Mancini, Claire Julian-Reynier, Catherine Noguès, Emmanuelle Mouret-Fourme, Betty Bouveur, Christine Lasset

En bas, de gauche à droite: Ariane Neveu, Noémie Resseguier, Anne-Déborah Bouhnik, Eve Bureau, Isabelle Pellegrini

UNICANCER GENETIC Group

SESSTIM UMR912 / Inserm / IRD Université AIX-MARSEILLE Sciences Économiques & Sociales de la Santé & Traitement de l'Information Médicale

Les recherches psychosociales ont reçu le soutien de l'Institut National du Cancer et de l'Agence de Biomédecine. La cohorte GENEPSO a reçu le soutien de la Fondation de France et bénéficie de soutiens renouvelés de la Ligue Nationale Contre le Cancer.

© UMR912 - GENEPSO - 2013 - septLieux.com

GENEPSO

GÈNE Prédisposition Sein-Ovaire

Un Programme de Recherche Médicale & de Sciences Humaines et Sociales

Résultats janvier 2013

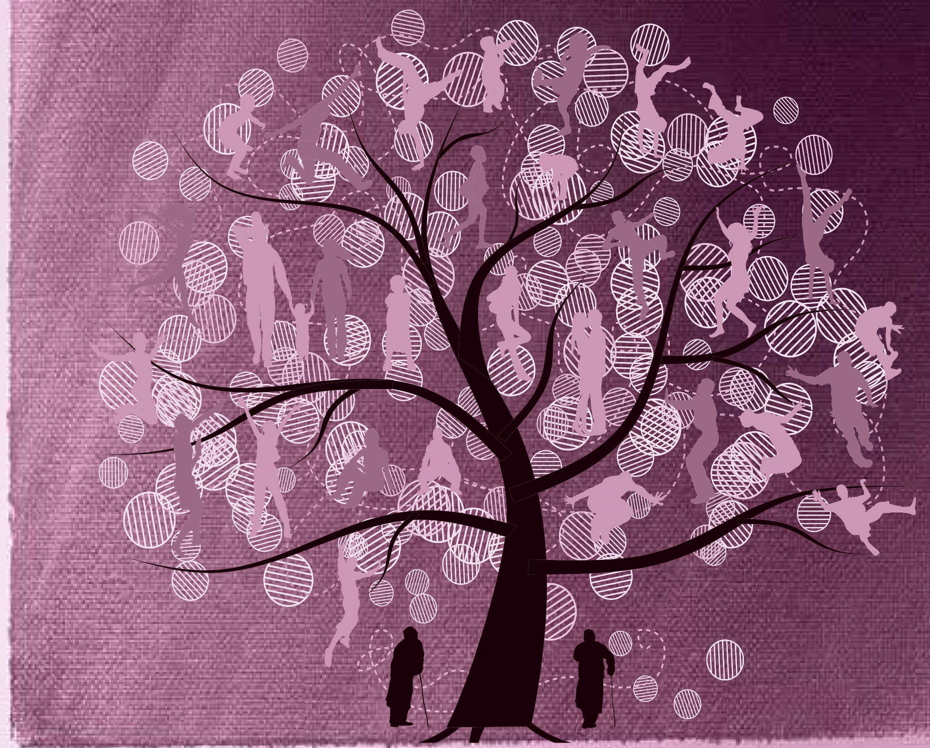

plaquette-photo.indd 1 27/03/13 16:06

GENEPSO : une cohorte* initiée en 2000

- 1600 familles différentes dans lesquelles une prédisposition génétique au cancer (mutation d'un des gènes *BRCA1/2*) a été identifiée.
- Plus de 2300 femmes et hommes porteurs d'une mutation d'un des gènes *BRCA1/2*, âgés de 18 à 98 ans, dont 83 % de femmes.
- Une cohorte de 363 femmes sans mutation.
- Des participants :
  - Progressivement inclus depuis 2000.
  - Venant de la France entière.
  - Suivis pendant 10 ans par des questionnaires de santé et des questionnaires psychosociaux.

32 centres 54 médecins investigateurs

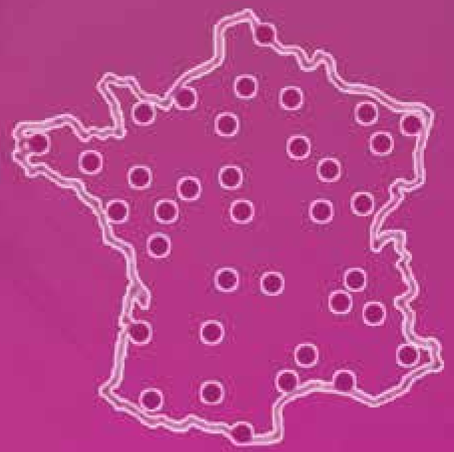

**** cohorte : population de sujets qui répondent à une définition donnée et qui sont suivis dans le temps***

Résultats de la recherche psychosociale

La participation à une étude par questionnaires

- La recherche par questionnaires est en général perçue de manière très favorable par les personnes qui ont participé. Elle constitue une source d'informations médicales au long cours. Cependant, elle peut soulever pour certains la question difficile du rappel de la maladie.

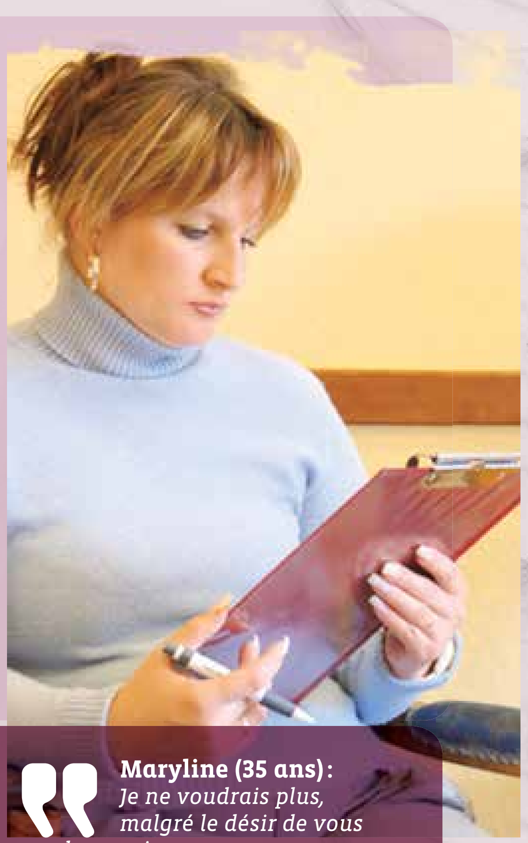

Marie-Jeanne (57 ans) : *Cela réconforte de recevoir votre courrier, je me dis que la recherche est nécessaire et profite à d'autres femmes…*

Maryline (35 ans) : *Je ne voudrais plus, malgré le désir de vous rendre service, par vos questionnaires, être replongée dans cette pathologie du cancer, que j'avais mise aux oubliettes…*

Les comportements observés de surveillance et de prévention

- Les femmes de moins de 40 ans porteuses d'une mutation *BRCA1/2*, optent plus souvent pour un suivi par imagerie mammaire (IRM* et mammographie) alors que les femmes de plus de 40 ans optent très souvent pour une ovariectomie préventive et une imagerie mammaire.
- La mastectomie préventive, qui est peu réalisée en France à l'heure actuelle, l'est plus souvent chez les femmes qui ont des enfants jeunes, et plus souvent lorsqu'elles sont déjà demandeuses de cette intervention avant les résultats des tests.
- L'ovariectomie préventive est réalisée par 8 femmes sur 10 de 50 ans et plus, dans les deux ans suivant les résultats des tests. Les femmes plus jeunes optent pour cette intervention de manière plus progressive en fonction de leur âge.

**IRM = Imagerie par Résonance Magnétique*

Les comportements observés de surveillance et de prévention chez les femmes non porteuses de mutation

- Après les résultats des tests, le plus souvent, les femmes sans mutation continuent à avoir un suivi de « femmes à risque », alors qu'au vu des connaissances actuelles, elles sont considérées comme ayant le même risque que les femmes de la population générale. Ce suivi spécifique n'est alors plus recommandé.

Agnès (32 ans) : *Malgré les résultats du test, au fond de moi, j'ai toujours la sensation d'être une personne à risque…*

Sylvie (41 ans) : *Je ne suis pas porteuse de la mutation, il n'empêche qu'il faut que je me fasse suivre comme une femme de mon âge…*

Les opinions vis-à-vis des diagnostics prénatal et préimplantatoire de mutation *BRCA1/2*

- Pour les jeunes générations de femmes et d'hommes porteurs de mutations *BRCA1/2*, le Diagnostic Prénatal et le Diagnostic Préimplantatoire sont des techniques suscitant un grand intérêt. Mais seule une minorité de ceux concernés directement par un projet parental souhaiteraient les utiliser pour eux-mêmes.

Émilie (29 ans) : *Oui, je peux le comprendre pour des couples qui veulent un enfant et ne souhaitent absolument pas lui passer la maladie, mais pas pour moi. J'ai eu la chance de voir ma mère qui a bien réagi à l'annonce de la maladie et pendant les traitements. Donc c'est une question de tempérament, d'histoire, de vécu…*

La diffusion du résultat du test à l'employeur

Valérie (31 ans) : *C'est moi qui l'ai annoncé à mon employeur dans l'objectif de me faire opérer et donc d'une absence de longue durée…*

- Alors que la loi ne les y oblige pas, un tiers des femmes ayant une activité professionnelle informent leur supérieur hiérarchique des résultats des tests génétiques, et ce, plus souvent lorsque leur niveau d'études est plus faible et lorsqu'elles ont décidé d'avoir une chirurgie préventive.

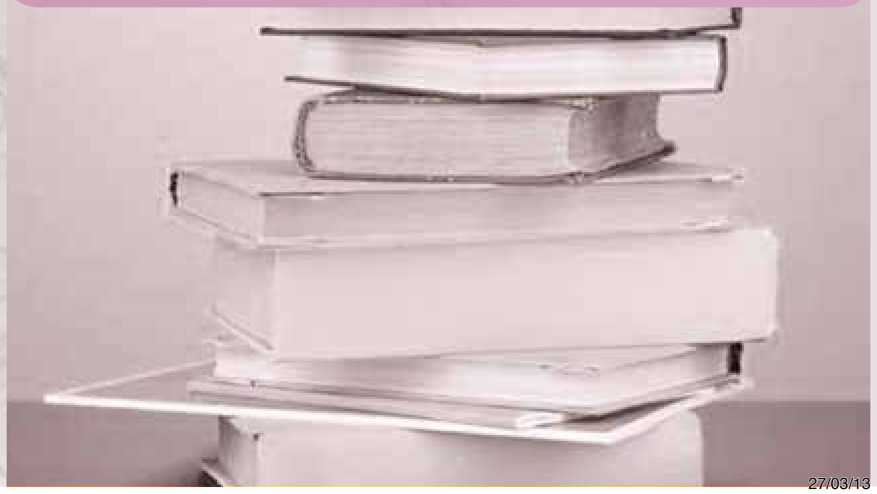

plaquette-photo.indd 2 27/03/13 16:07

Quelques actualités biologiques et médicales dans le domaine des gènes *BRCA1/2*

- Depuis l'identification des gènes *BRCA1* et *BRCA2* en 1994-95, plus de **2000** mutations de ces gènes ont été découvertes dans les laboratoires.
- À l'heure actuelle, un suivi par **IRM*** mammaire est proposé dans le cadre de la surveillance **annuelle** par mammographie et échographie mammaire à partir de l'âge de **30 ans** chez les femmes porteuses d'une mutation.
- La mastectomie préventive permet une réduction de plus de **90 %** de la survenue des cancers du sein.
- L'ovariectomie préventive permet une réduction de plus de **90 %** de la survenue des cancers de l'ovaire, mais aussi une réduction de la survenue des cancers du sein.
- Une meilleure connaissance des tumeurs qui se développent chez les personnes ayant des mutations *BRCA1/2* permettra, en fonction de nouvelles recherches, d'adapter de mieux en mieux leur traitement ou de proposer des méthodes nouvelles de prévention.
- Quelques **actualités** sur l'organisation de l'oncogénétique **en France** :
  - Activité soutenue depuis **2003** par les **Plans Cancer**
  - Plus de **110** lieux de consultations
  - Plus de **60 000** tests de prédisposition génétique aux cancers du sein et de l'ovaire réalisés depuis 2003
  - Plus de **12 000** personnes porteuses de mutations *BRCA1/2* dont à peu près **20 %** sont incluses dans la cohorte **GENEPSO**

** Imagerie par Résonance Magnétique*

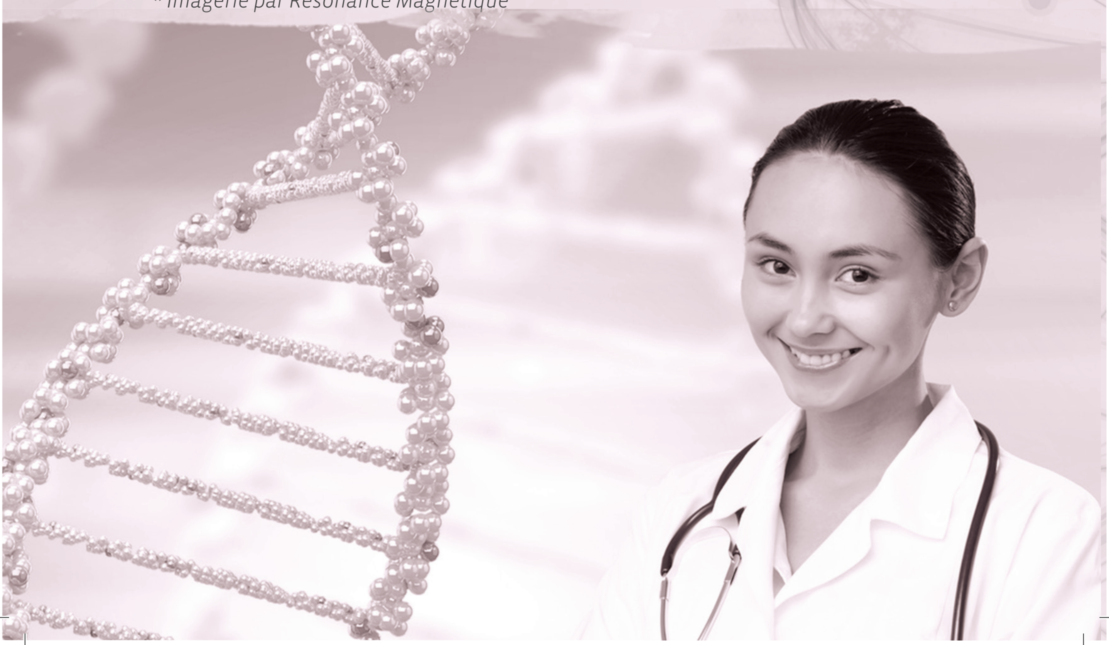

Feuillet 1 27/03/13 16:03

Le programme GENEPSO :

Des participants, un réseau collaboratif de médecins (Groupe Génétique et Cancer), des chercheurs, associés pour un même objectif collectif de progression des connaissances.

Pour la recherche en Sciences Humaines et Sociales, des équipes de recherche coordonnées :

Par le Docteur Claire Julian-Reynier (Institut Paoli-Calmettes) & le Docteur Catherine Noguès (Institut Curie)

Des compétences de multiples disciplines de recherche :

Médecine, génétique, épidémiologie, biostatistiques, psychologie, anthropologie, économie, sociologie.

UNICANCER GENETIC Group

SESSTIM
UMR912 / Inserm / IRD
Université AIX-MARSEILLE
Sciences Economiques & Sociales de la Santé & Traitement de l'Information Médicale

© UMR912 - GENESPO - 2013 - septLieux.com

Les recherches psychosociales ont reçu le soutien de l'Institut National du Cancer et de l'Agence de Biomédecine. La cohorte GENEPSO a reçu le soutien de la Fondation de France et bénéficie de soutiens renouvelés de la Ligue Nationale Contre le Cancer.
